# Design of High Speed and Low Offset Dynamic Latch Comparator in 0.18 µm CMOS Process

**DOI:** 10.1371/journal.pone.0108634

**Published:** 2014-10-09

**Authors:** Labonnah Farzana Rahman, Mamun Bin Ibne Reaz, Chia Chieu Yin, Mohammad Alauddin Mohammad Ali, Mohammad Marufuzzaman

**Affiliations:** 1 Department of Electrical, Electronic and Systems Engineering, Universiti Kebangsaan Malaysia, Bangi, Selangor, Malaysia; 2 MIMOS Berhad, Technology Park Malaysia, Kuala Lumpur, Malaysia; Gazi University, Turkey

## Abstract

The cross-coupled circuit mechanism based dynamic latch comparator is presented in this research. The comparator is designed using differential input stages with regenerative S-R latch to achieve lower offset, lower power, higher speed and higher resolution. In order to decrease circuit complexity, a comparator should maintain power, speed, resolution and offset-voltage properly. Simulations show that this novel dynamic latch comparator designed in 0.18 µm CMOS technology achieves 3.44 mV resolution with 8 bit precision at a frequency of 50 MHz while dissipating 158.5 µW from 1.8 V supply and 88.05 µA average current. Moreover, the proposed design propagates as fast as 4.2 nS with energy efficiency of 0.7 fJ/conversion-step. Additionally, the core circuit layout only occupies 0.008 mm^2^.

## Introduction

An analog-to-digital converter (ADC) is the vital component to drive the integrated circuit (IC) design industry in recent years. Portable electronic systems such as devices used in the wireless communication, consumer electronics or medical equipments elevates the requirement of producing low–power, high speed circuit methods and building blocks. As a result, integrating various functional blocks inside an IC makes ADCs more traditional to generate high speed and low power consumption. However, few characteristics of ADCs as reduced transistor sizes, low power dissipation, decreased propagation delay make it more suitable to the recent IC industries [1]. Therefore, to be compatible with the above-mentioned features, a new ADC with decreased supply voltage according to the transistor dimension is needed, where a major building block is the comparator [2].

Comparator is the key building block in the design process of ADC, which controls the performance and the accuracy of ADCs. Switching power regulators, data receivers, memory circuits, radio frequency identification (RFID), etc. requires high-speed, high-resolution and low-power comparators [3]–[4]. Therefore, high performance comparators are necessary to amplify small input voltage to a big output voltage. Consequently, a faster and accuracy based comparator involves high gain and high bandwidth [5]–[6].

Several high-speed comparators like multistage open loop comparator; the regenerative latch comparator and the preamplifier latch comparator exits, which are most accepted configurations [7]. For many applications, CMOS dynamic latch comparators are very popular due to fast-speed, low-power consumption, high-input impedance and full-swing output. Moreover, latch type comparators are capable of generating higher gain in regeneration mode using positive feedback mechanism. However, to design the latch comparator for low voltage operations, which are capable of reducing the dynamic input ranges and the analogous differential process sometimes elevates the power indulgences in rail-to-rail maneuver [2], [8]. However, the accuracy of the latch type comparators can be decreased by the random offset voltages generated due to device mismatches and random noise. As a result, decreasing offset voltages is one of the vital design parameters to design the dynamic latched comparator [9].

Conventionally, to decrease the offset voltage, a pre-amplifier has been utilized prior to the regenerative latch stage, which is able to amplify a small input signal to a large output signal to conquer the latch offset voltage and the kickback noise [6], [10]. Nevertheless, a pre-amplifier based comparator performance is affected from large static power dissipation. Therefore, a dynamic latch comparator without pre-amplifier is very much enviable for high speed and low power applications. At present, dynamic latch comparators with offset cancellation methods has been proposed. Verma and Chandrakasan proposed such a novel structure of offset calibration latch comparator [11]. However, the proposed design is not suitable for high-speed applications due to a large number of offset cancellation capacitors. In 2008, a dynamic comparator with a charge pump circuit is proposed to order related input referred offset voltage, which makes the approach inefficient [12]. However, the involvement of this charge pump circuit limits its accuracy.

In this research, a novel dynamic latch comparator is proposed, which is based on differential pair input stages along with one cross-coupled stage. The proposed design is capable of generating high-speed, high resolution with low power indulgence in low supply voltages compared to the conventional dynamic latch comparators. Moreover, the proposed design provides a small layout area than the other research works. In this design, Cadence Virtuoso 0.18 µm CMOS process is employed for the pre-layout and post-layout simulation. The simulated results show that, the circuit topology makes it valid to perform under low supply voltage.

### Background

**Figure 1:**
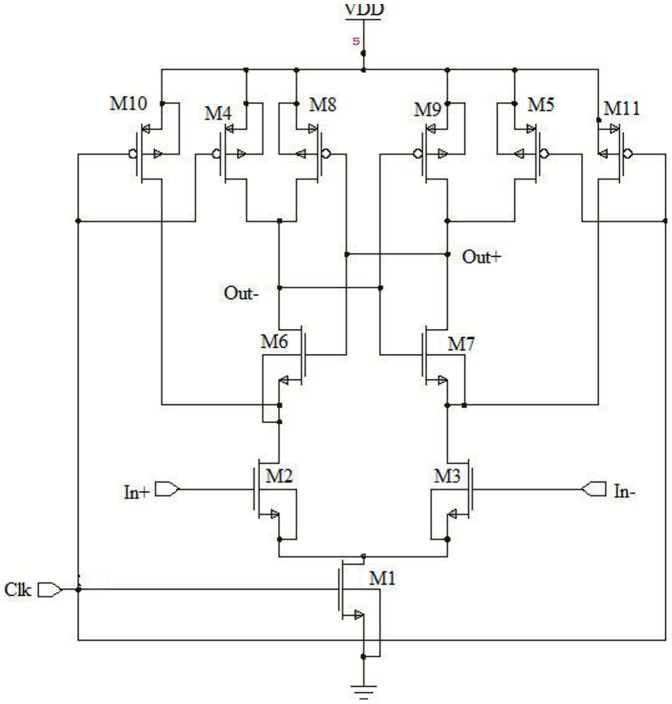
Conventional dynamic latch comparator [13], [14].

Figure 1 show the conventional dynamic latched comparator, which is most widely used due to its high input impedance, zero static power, high-speed and full swing output [13]–[14]. In the architecture of the Kobayshi et al. and Wicht et al., only transistor M1 exist at the tail, which controls the current flow between the differential pair input M2 and M3 and the latch formed by M6–M9. It is unavoidable to resize the transistor M1 in order to rise up the current flow through the latch. At the evaluation phase, when Clk  =  VDD, the drain current of the two input transistors (M2 and M3) is increased according to the increment of the size of M1 transistor.

**Figure 2:**
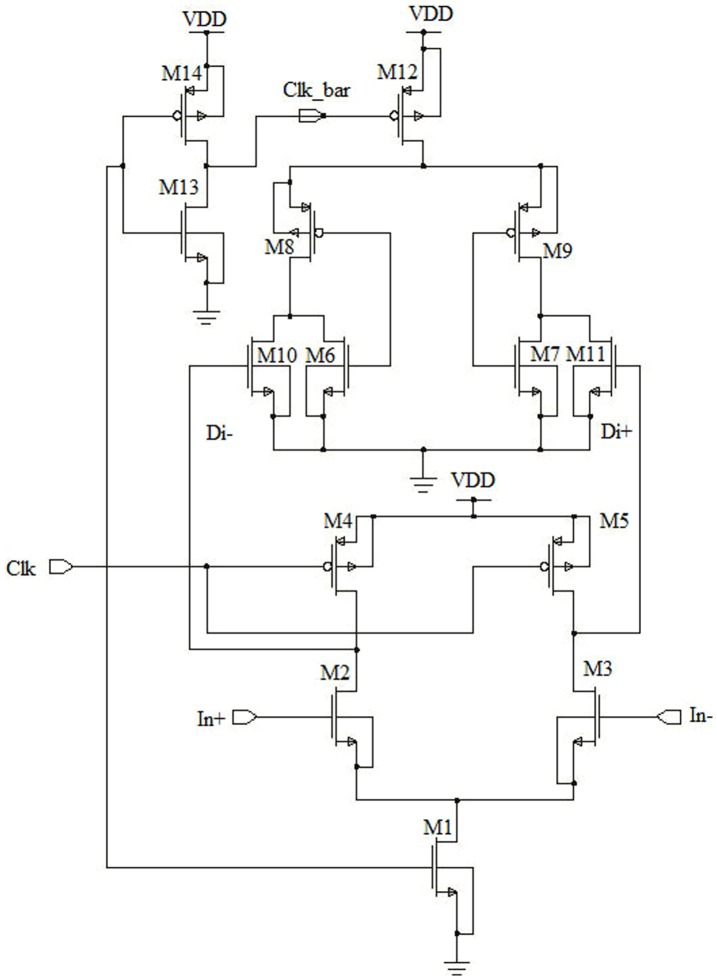
Double-tail latch-type voltage sense amplifier.

Therefore, the time length of the saturation region of the input transistor reduced, when the Di node discharged from VDD to ground in a very small period. In addition, a large input offset voltage exists due to the mismatch in threshold voltage (V_th_) between M6 and M7 and low amplification of the input voltages around Di nodes. Moreover, it is not popular for applications like ADCs, which requires a wide input common mode range as it has a contrasting values in speed and offset voltage [14]–[15]. All these limitations has been circumvented by the design of Schinkel et al. as shown in Figure 2, which consist of double-tail latch type sense amplifier [15].

**Figure 3:**
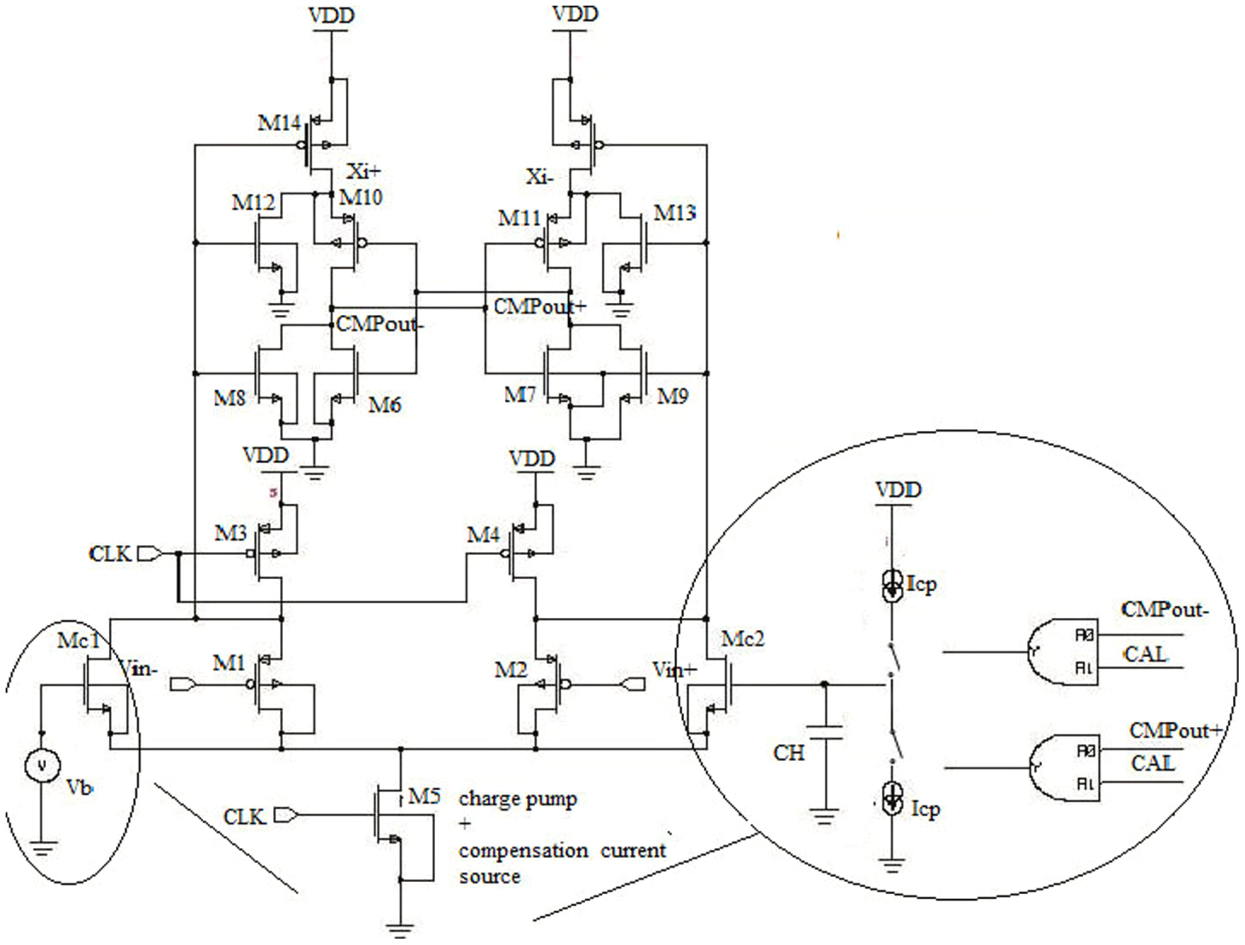
Self-calibrating comparator.

The designed circuit is capable of working under a low supply voltage, as the comparator architecture is separated with stable offset voltage and faster input common-mode voltage range (Vcom). However, the architecture requires both the clock signals (Clk and Clkb) with precise timing correlation among the clock signals for best possible operation. At the evaluation phase, when Clk  =  VDD and there is jitter on this rising edge the formation of voltage difference occurs between the Di nodes and this variation affects the speed and the offset voltage. This variation is happened due to clock skew between the clock signals. For a small delay, Clk signal is able to drive auxiliary inverters, which coerce the M2 transistor by adding a simple inverter to produce the Clkb signal. It results a larger delay Clkb, which is lagging behind the Clk signal. On the other hand, due to short circuit path among the M12, M10, M11, M8 and M9 transistors the power dissipation is increased. Miyahara et al. proposed a comparator as shown in Figure 3 with the calibration method, where Clkb is replaced with Di nodes to solve the clock skew problem [11].

This technique resolved the limitation of reduced clock load due to the skew problems of the clock signals. Moreover, noise and input offset voltages are decreased as the circuit employed double transconductance (g_m_) in the Di nodes with larger node capacitances. However, the circuit is affected by the increased delay as the current drivability of the output load becomes weakened.

**Figure 4:**
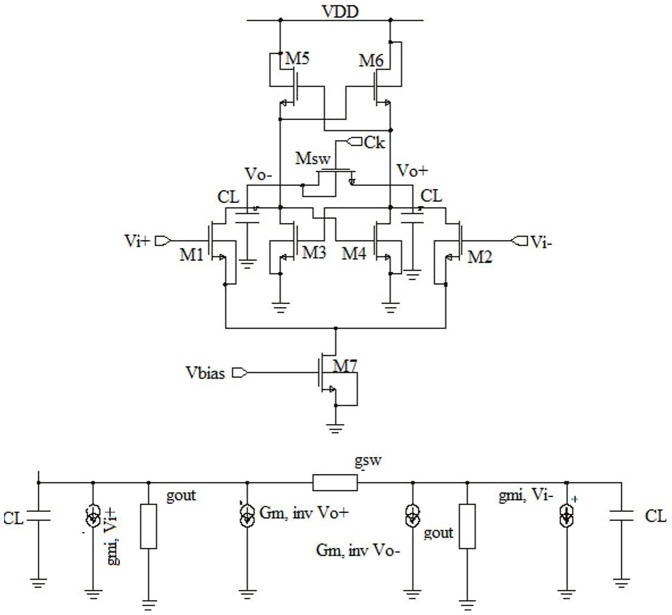
Schematic diagram of the track and latch comparator (a) conventional track and latch comparator (b) small signal model of the comparator.

The track and latch comparator structure is shown in Figure 4. Figure 4 is subdivided into two different blocks, where Figure 4(a) design is constructed with an input differential pair; a regenerative latch and a regeneration control switch [16].

The operation method is also subdivided into two modes. By adding an extra low resistive load on the output node in the track/first phase, Msw switch becomes on as the CK signal is high, which eventually prevents the regeneration of the latch. By choosing a proper drain-source conductance of Msw, which is compared with the transconductance of the inverters, the positive feedback of the latch is ceased. As a result, to step up for the next phase/mode, the input signals are tracked by the output node voltage and latch is initiated for appropriate regeneration. In the latch/second phase, Msw transistor is turned off with the CK signal going low to start the regeneration and to toggle the output with positive feedback. If the initial voltage at output node becomes higher than the latch offset voltage, then the latch makes correct decision. The small signal model of the latch comparator is shown in Figure 4(b). The output voltage can be written as:
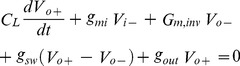
(1)

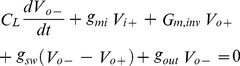
(2)where, *g_mi_* is the input differential pair transconductance, *G_m,inv_* is inverter transconductance, *g_sw_* is the drain–source conductance of M_sw_ switch, *g_out_* and *C _L_* are the equivalent conductance and capacitive load at output nodes, respectively. Equation (1) and (2) can be simplified for the symmetric condition of the circuit devices as:
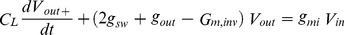
(3)where, (V_out_  =  (V_0+_ - V_0−_)) and (V_in_  =  (V_in+_ - V_in−_)). The above-mentioned equation can be solved with the assumption of constant or slow variant input signal is as follows:



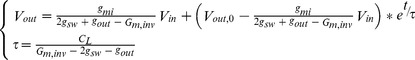
(4)From equation (4) it is found that, the initial voltage of *V_out_* is defined as *V_out,0_*  =  time constant of latch in the output node. The value of *s* is varied according to the load capacitance and difference of inverters transconductance and equivalent conductance at output node. If the value of 2*g_sw_* + *g_out_* ≥ *G_m,inv_* then the time constant is found negative and the second term in equation can be negligible. Moreover, the output node voltage is tacked according to the input of track mode operation of latch process. On the other hand, if *G_m,inv_* ≥2*g_sw_* + *g_out_* the time constant is found positive and the exponential regeneration in output node is found from the initial value in regeneration mode. For successful regeneration process, it is required to get the correct decision of getting larger initial voltage on latch output node. From Figure 4 it is also found that, to solve the problem of offset voltage in high speed and high-resolution applications, pre-amplifier stage is added before latch stage.

**Figure 5:**
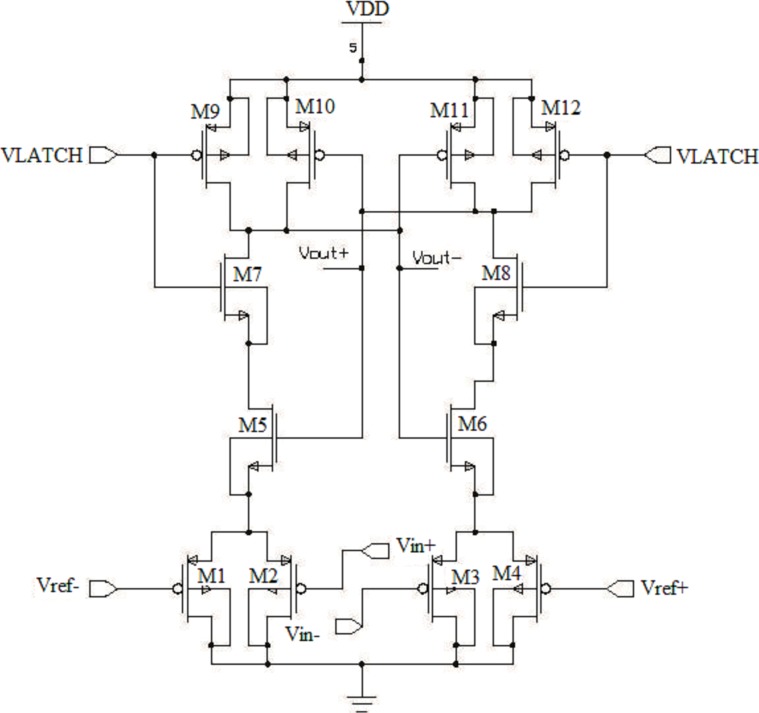
Lewis-Gray comparator.

Generally, static random access memories (RAMs) utilized the most commonly used dynamic comparator. This comparator is mainly used for pipelined ADCs, which are structured from the differential sensing amplifier. Cho et al. proposed a dynamic comparator known as, ‘Lewis-Gray’ dynamic comparator is shown in Figure 5 [17]. The main advantage of this design is the zero DC power dissipation and built –in circuitry to adjust the threshold voltage.

In Figure 5, to adjust the threshold voltage, transistors M1–M4 is utilized as the adjustment circuitry. On the other hand, latch circuit is composed with the transistors M5–M12. M1–M4 transistors are used as the adjustment circuitry for the threshold voltage and transistors M5–M12 works as a latch, which is shown in Figure 1. When, V_latch_  = 0 V, transistor M9 and M12 is in the conducting mode, M7 and M8 go to cut off region. As a result, both the differential outputs become V_dd_ and there is no current passes between the supply voltages. Concurrently, M10 and M11 are in cut off region while M5 and M6 are in conducting mode. As a result, V_dd_ exist a voltage over M7 and M8. On the other hand, when V_latch_  =  V_dd_ both the transistor M7 and M8 turns on but keeps M5 and M6 transistors in saturation region as both of these transistors gates still holds the value V_dd_. The inputs of the left and right branches formed by M1–M4 determines whether the output holds the value V_dd_ or 0 V, which is mainly depends on the mismatch of the transistors M5–M12. In a static situation when, V_latch_  =  V_dd_, the outputs holds the values until the comparator is reset to 0 V as both the branches are in cut off region. The inputs *V^+^_ref_* and *V^−^_ref_* are in triode region and acted as voltage controlled resistors. If the transistors are fully matched (W_A_ = W_2_ = W_4_ and W_B_ = W_1_ = W_3_) and the conductance of the left and right branches are equal i.e. *g_L_* = *g_R_* then the comparator changes its outputs as shown in equation (5).
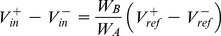
(5)


Equation (5) implies that, the mismatch of the transistor M1–M4 can create the offset and the condition is true if all other transistors M5–M12 are matched properly. Whereas, the transconductance of M1–M4 and M5, M6 can be written with the following equations (6) and (7):
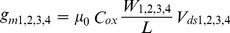
(6)

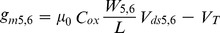
(7)where, threshold voltage  = V_gs5,6_, corresponding gate-source voltage of M5 and M6 = *V_gs5,6_*. In initial latching phase *V_ds1,2,3,4_* = 0 while,*(V_gs5,6_ - V_T_ = V_dd_)*. To determine the latching balances, the magnitude of the transconductances of *g_m5,6_* should be higher than *g_L_ and g_R_* of the input branches. If there is any mismatch then it will create an offset voltage. To minimize the power dissipation and the size of the area, all the transistors are set with minimum sizing, which results offset voltage with few hundred mV. Moreover, the mismatch in input transistors is amplified by the M5 and M6 transistors. In addition, any mismatch in M7–M12 transistors are attenuated by the gain of M5 and M6. To solve the mismatch problem, layouts of the critical transistors are drawn symmetrically. Moreover, the load capacitances are circumventing by additional latch or inverters, which works as a buffer step after the core outputs of the comparators.

## Materials and Methods

**Figure 6:**
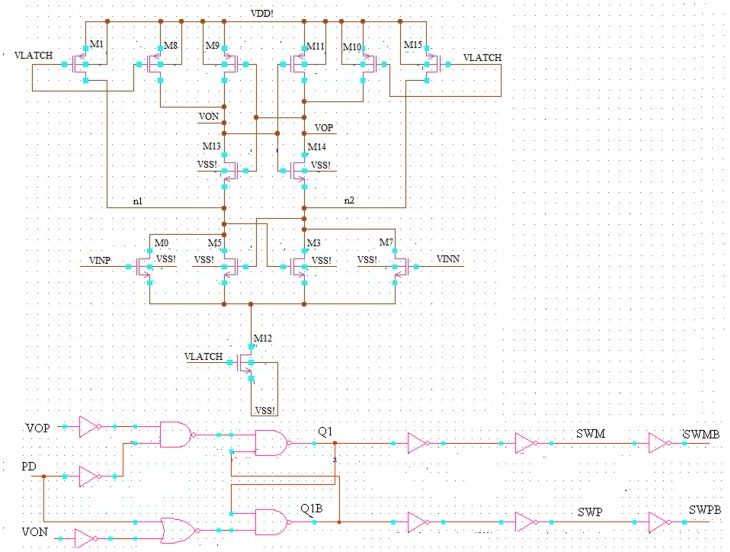
Schematic diagram of the proposed differential pair dynamic latch comparator with S-R flip-flop.

Offset voltage is the main limitation of designing a dynamic latch comparator. As a result, offset voltage can be reduced or cancelled with proper transistor sizing/matching during the design process against mismatch and process variation. In this research, a fully differential dynamic latch comparator is presented inspired by the design of ‘Lewis-Gray’ dynamic comparator, which is based on cross-coupled differential pairs as shown in Figure 6 [17].

The proposed design consists of transistors M0, M3, M5 and M7 as the input circuitry, which is depicted, in Figure 6. To configure the overall latch circuitry, M1, M8, M9, M10, M11, M12, M13, M14 and M15 transistors have been included in the proposed design. In this proposed topology, the supply voltage is passed through M1, M8, M9, M10, M11 and M15 transistors and the latch circuit is directly attached with the source coupled pairs M3 and M5, which provides the switchable current sources. Regenerative latch configurations of S-R flip-flops are also utilized, where a power down (PD) is added to reset the S-R latch at the output of the comparator. The operation of the proposed comparator is divided into two phases. In the first stage, when, the comparator is off and VLATCH  =  “0”, no current path exists from the supply voltage as the current source M12 is switched off. In this case, all the PMOS transistors M1, M8, M10 and M15 reset the output VON and VOP and node n1 and n2 to VDD. On the other hand, when VLATCH  =  “1”, the outputs are disconnected from the positive supply. In addition, switching current source M12 begins to conduct, which decides the bias current of the input transistors M0, M7, M3 and M5. To generate positive feedback to allow the faster switching at the output, cross-coupled NMOS pair M3 and M5 is utilized in the proposed topology. However, inputs VINP and VINN determine the switching. In addition, the output signals SWM and SWP remains unchanged (combine with above). When the voltage VINP > VINN, the drain voltage of M0 started to fall at a faster rate than the drain voltage of M7. At this point, once the positive feedback from the cross-coupled NMOS transistors M5 and M3 kicks in, the node n1 drops even faster and pull node VON to low. As a result, a logic low at the RS latch is created and the output Q becomes low. The overall transistor dimensions decide the overall circuit performance, which is shown in Table 1.

**Table 1:** Transistor dimensions used in this proposed topology.

	Lewis-Grey		Proposed Comparator	
Transistors	W(µm)	L(µm)	W(µm)	L(µm)
M0	x	x	4	2
M1	5	1	2	0.18
M2	20	1	x	x
M3	20	1	4	2
M4	5	1	x	x
M5	3	0.5	4	2
M6	3	0.5	x	x
M7	3	0.5	4	2
M8	3	0.5	2	0.18
M9	8	0.5	2	1
M10	8	0.5	2	0.18
M11	8	0.5	2	1
M12	8	0.5	2	0.18
M13	x	x	1	1
M14	x	x	1	1
M15	x	x	2	0.18

As mentioned earlier, the offset voltage depends on the mismatch of the threshold voltage ΔV_T_, load resistance ΔR_L_ and transistor dimensions Δβ and the corresponding average values (V_T_, R_L_, β), which can be given by the following equation.
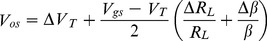
(8)


In equation (8) the offset voltage is dominated by the Δβ, which is the mismatch of the transistor dimension and the overdrive voltage *V_gs_ - V_T_*. From this equation, it is found that, if the common mode voltage becomes lower *V_gs,low_*, then offset voltage is found smaller. However, the effect of the mismatches of the transistor (M1, M8, M9, M10, M11, M13, M14 and M15) found from the simulations are not very critical. Moreover, all the transistors used as the input differential pair and the cross coupled NMOS are important as all these M0, M3, M5, M7 and M12 transistors settled the overdrive voltage of the input differential pair, which is proportionally related to the *V_gs_ - V_T_* as shown in equation (8).

## Results and Discussion

**Figure 7:**
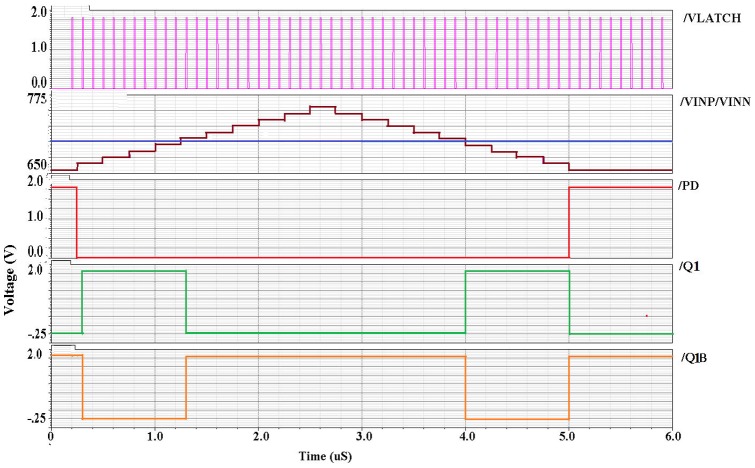
Transient simulation of the comparator input signals, VLATCH signal and output signals (SWP and SWM) using Virtuoso Spectre.

The proposed dynamic latch comparator circuit has been verified using SPECTRE simulator (CADENCE). CADENCE Virtuoso in a 0.18 µm CMOS process parameter is utilized in this design. The simulated behavior of the comparator is illustrated in Figure 7. It is observed from Figure 7 that, with a 2 mV positive step size for the input VINP and keeping VINN fixed at 0.7 V the proposed dynamic latch comparator can switch successfully. In this topology, the stepping of the input signal VINP (going up and coming back down) is used to check whether there is any hysteresis or not [18]. From the simulated results of Figure 7 it is found that, when VINP > VINN on the rising edge of the VLATCH signal, the output Q1 and Q1B switches successfully. Similarly, switching also happens whenever, the VINP < VINN during VLATCH =  high.

**Figure 8:**
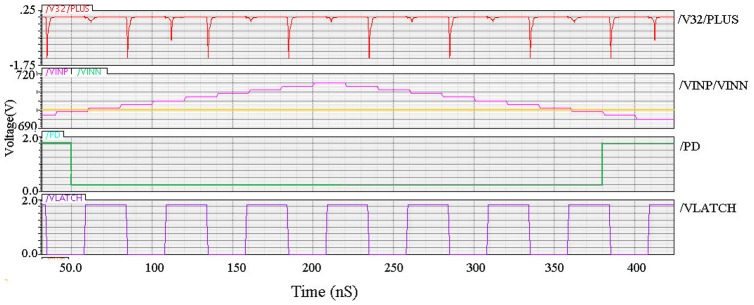
Post-layout simulation results for average current of dynamic latch comparator.

In this research, the proposed dynamic latch comparator consumes lower current, which is shown in Figure 8. From the simulated result, it is found that the proposed latched comparator requires only 88.05 µA, which is average current for 50 MHz clock frequency. Moreover, the simulated result shows that the power dissipation for the proposed dynamic latch comparator is 158.5 µW under 1.8 V supply voltage.

**Figure 9:**
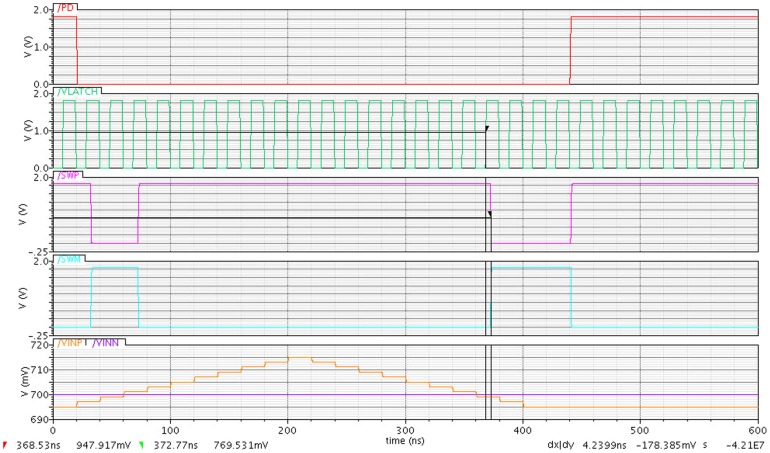
Propagation delay waveform between the VLATCH and SWP signal.

The propagation delay is one of the significant factors in designing a dynamic latch comparator. Generally, the propagation delay is inversely proportional to the input voltage applied. Moreover, a larger input voltage applying will increase the propagation time and creates more delay [19]. The propagation delay sets the maximum frequency of operation for the latch. In this design, the proposed dynamic latch comparator propagates as fast as 4.2 nS as shown in Figure 9. In addition, this small delay makes the clock or latch signal of the proposed design faster up to 50 MHz.

**Figure 10:**
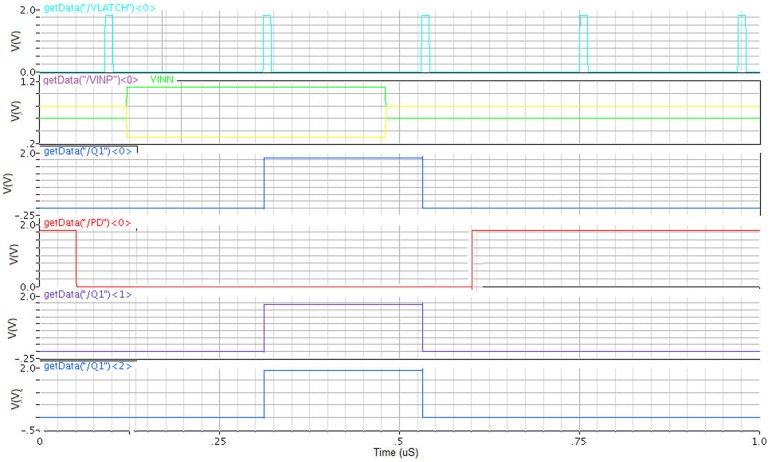
Corner analysis of the comparator input/output signals.

In analog design, manufacturing tolerances for devices, temperature range and variations of external signals are verified by corner analysis and process variation simulations. To test the proposed dynamic latch comparator all 45 corners 3 Vcc (1.7 V, 1.8 V and 1.9 V), 3 temperature and 5 corners are analyzed with different stepping size of VINP and a fixed VINN at 0.7 V, which is shown in Figure 10. The corner test results revealed that, the dynamic latch comparator is able to switch properly at different corners of VDD and temperature.

**Figure 11:**
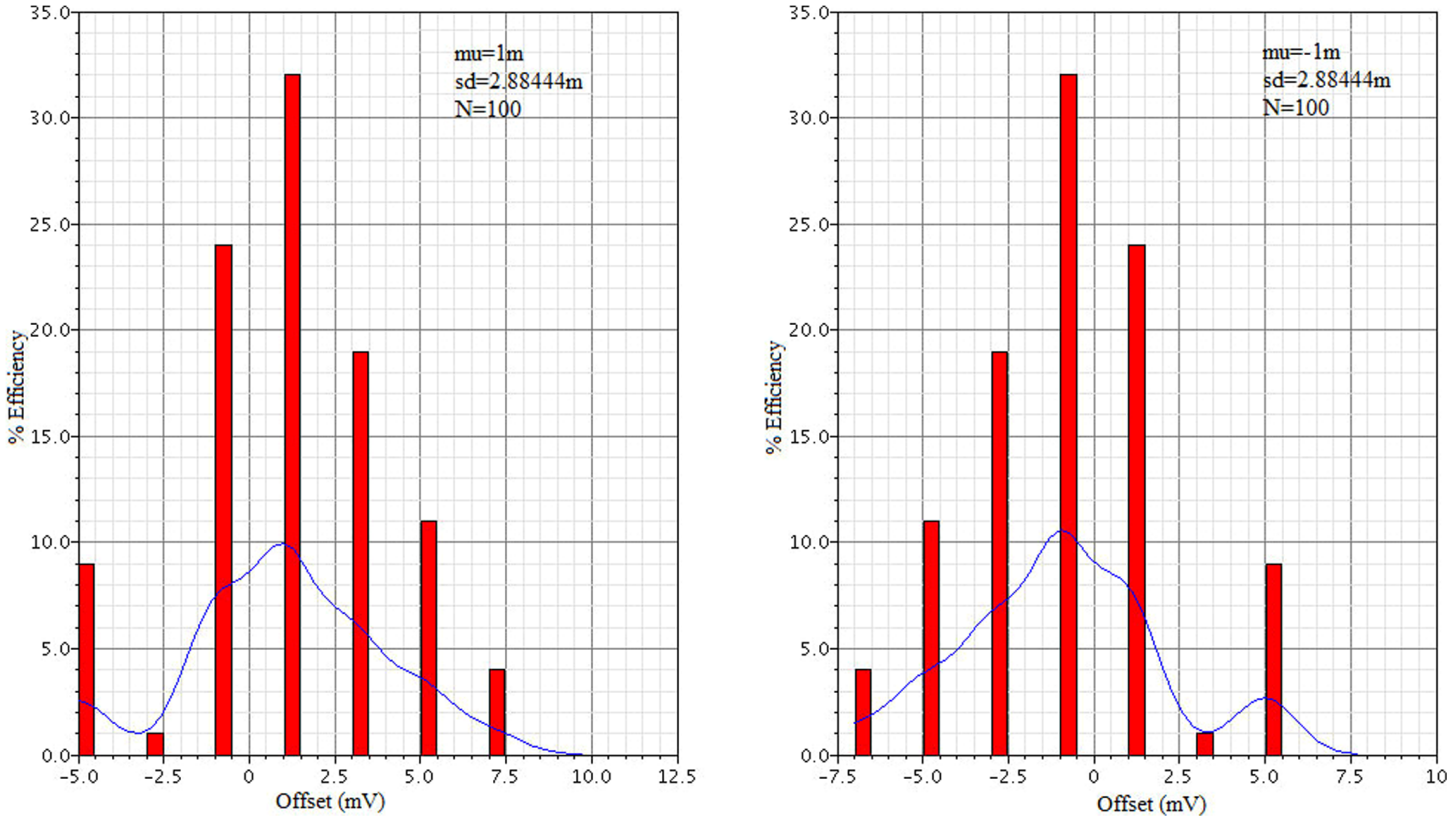
Pre-layout Monte-Carlo simulation result for the proposed design.

To calculate the impact of transistor mismatch a Monte-Carlo simulation with 100 runs was performed. This evaluation is also gives the process variation mismatch. The results, shown in Figure 11, reveal that the designed comparator generates an average offset voltage of 1 mV with 2.88 mV standard deviation.

**Figure 12:**
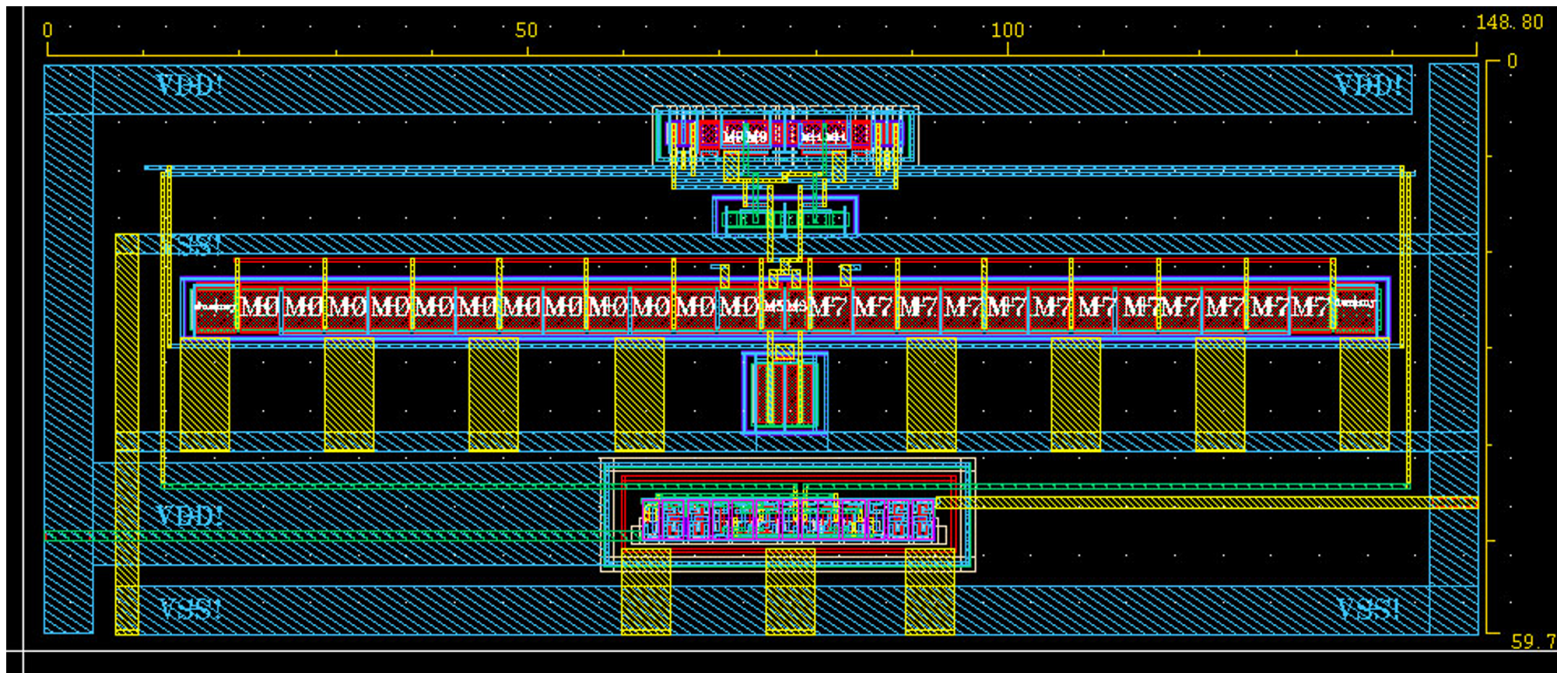
Core circuit layout diagram of the proposed dynamic latch comparator.

The chip layout is shown in Figure 12 where the chip occupies a small area of 148.80 µm ×59.70 µm. During the design process, all the transistors are placed symmetrically to reduce mismatch and parasitic capacitance.

**Figure 13:**
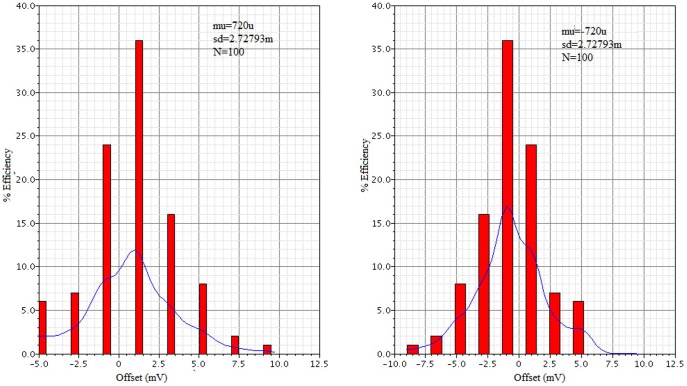
Post-layout Monte-Carlo simulation of the proposed dynamic latch comparator.

The post-layout Monte-Carlo simulation results for 100 runs is shown in Figure 13, which found that a higher offset value was obtained at a sampling frequency of 50 MHz using VDD 1.8 V with the overdrive voltage of 3.44 mV, which corresponds to 0.5 LSB at 8-bit precision.

Table 2 summarizes the performance of the proposed dynamic latch comparator along with other research works. Generally, offset voltage results from device mismatch. Compared to [20] the proposed dynamic latch comparator has less offset voltage. In this research, propagation delay sets the maximum frequency of operation for the latch signal of the proposed dynamic latch comparator. As a result, the proposed latch comparator is able to propagate as faster as 4.2 nS, which is significantly lower than [20] and [23]. In addition, this small delay makes the clock or latch signal of the proposed design faster up to 50 MHz, which makes the proposed design superior for high speed applications like ADCs than [21], [22], [23] and [24]. From the comparison Table 2 it is found that, the proposed design has a small layout area than [21] and [22]. However, the core layout area of the proposed design is similar to [20] and [23]. Moreover, the proposed design dissipates lower power compared with [22] and [24].

**Table 2:** Performance comparison of the proposed dynamic latch comparator.

References	[20]	[21]	[22]	[23]	[24]	This Work
Technology (µm -CMOS)	0.5	0.35	0.18	0.18	0.6	0.18
Supply Voltage (V)	±1.25	1.2	1.8	1	-	1.8
Reference Voltage(mV)	-	0.6	0.8	-	-	0.7
Power P_d_(µW)	-	-	225	63.5	750	158.5
Average Current (µA)	3.1	125	-	-	-	88
Sampling Rate (MHz)	-	20	30	20	40	50
Resolution (bits)	-	8	8	12	8	8
Propagation delay (nS)	932^a^	-	-	26^a^	-	4.2
Offset voltage (mV)	24.2	3	-	0.0476	-	3.44
FOM (fj/conv)	-	1.64	15.7	0.77	73.2	0.7
Layout area(mm^2^)	0.086	0.021	0.016	0.008	-	0.008

a Measured value.

To compare the performance of different comparators, a well-known figure of merit (FOM) is used [25]. Therefore, in this research, to measure the performance of the design, the FOM is calculated using the following equation:
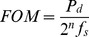
(9)where, *P_d_*, is the power indulgence, *n* is the number of bits (resolution), and *f_s_* is the sampling frequency of the comparator. From the comparison study of different research works shown in Table 2, the proposed dynamic latch comparator has the lowest FOM energy dissipated per conversion.

In this research, the proposed dynamic latch comparator is able to work for 8-bit resolution, whereas the resolution for the comparator proposed in [23] is found to be 12-bit. To improve the overall resolution of the proposed comparator, different transistor sizing and layout methods in the realized chip can be implemented. Moreover, the proposed design has removed fully the preamplifier stage and has a dynamic latch, resulting in a significant power saving, especially in flash and pipelined A/D architectures and RFID transponders.

## Conclusions

A novel high-speed, low power and low offset dynamic latch-type comparator method is presented in this research works. The proposed design does not use any preamplifier stages before latch stage, which eventually reduces the power dissipation and the area dramatically. The corner analysis and the Monte-Carlo simulation results clearly reveal that, the dynamic latch comparator is able to switch properly with different input stepping sizes. Moreover, the comparison study shows that, the novel design is able to operate at a higher clock frequency of 50 MHz with lower offset voltage 3.44 mV and propagation delay 4.2 nS, which is better than recently published research works. Moreover, the proposed design consumes 158.5 µW of power from 1.8 V supply and 88.05 µA average current. The core circuit layout occupies only 0.008 mm^2^, which makes the proposed design superior than the previous research works.
